# Assessing community-acquired acute infection in an irish emergency department: A retrospective observational study protocol

**DOI:** 10.1371/journal.pone.0359954

**Published:** 2026-10-05

**Authors:** Nandakumar Ravichandran, Etimbuk Umana, Tomas Breslin, Aoife Cotter, Walter Cullen

**Affiliations:** 1 School of Medicine, University College Dublin, Dublin, Ireland; 2 Mater Misericordiae University Hospital, Dublin, Ireland; Christian Medical College Vellore, INDIA

## Abstract

Sepsis is a life-threatening condition associated with substantial morbidity and mortality worldwide. In Ireland, the majority of sepsis cases are community-acquired; however, patients presenting to emergency departments (EDs) with suspected severe infection remain poorly characterised, particularly those not captured in administrative datasets. This retrospective cross-sectional observational study will be conducted in the ED of Mater Misericordiae University Hospital, Dublin. Adult (non-maternity) ED presentations across eight predefined 24-hour sampling periods will be reviewed. Community-acquired acute infection will be identified through manual review of ED clinical documentation, and possible sepsis will be identified using the ED triage sepsis discriminator supported by clinical documentation. Data collected will include demographic characteristics, referral pathways, clinical presentation, and patient outcomes. Multivariable logistic regression will be used to identify factors associated with adverse outcomes, defined as intensive care unit admission or in-hospital mortality. This study will provide a detailed evaluation of ED presentations with suspected severe infection in Ireland, including patients not captured in existing national datasets. Findings will contribute to improved understanding of early sepsis recognition, referral pathways, and resource planning in emergency care.

## Introduction

Sepsis is a life-threatening organ dysfunction resulting from a dysregulated host response to infection [1]. It represents a time-critical medical emergency, yet it remains difficult to recognise, particularly in primary care settings. Without prompt detection and management, sepsis can rapidly progress to tissue damage and multi-organ failure. According to the National Sepsis Report (2023), sepsis and septic shock were documented in 14,535 non-maternity adult patients in Ireland, with a mortality rate of 20.6% (2). Community-acquired sepsis represents a substantial public health burden, with over 70% of all sepsis cases originating in the community rather than hospitals [2–4].

A recent Irish study examining sepsis between 2016 and 2022 identified 86,011 community-acquired sepsis (85.2%) and 14,930 hospital-acquired (14.8%) episodes, with hospital-acquired sepsis defined as infection onset occurring at least two days after admission [5]. Socioeconomic disparities were evident, with patients from lower socioeconomic backgrounds demonstrating a 20% higher likelihood of death.

General practitioners (GPs), GP-out-of-hours services, paramedics, and community healthcare professionals are often the first point of contact for patients with severe infection [6]. Irish general practice facilitates over 29 million consultations annually, in addition to alongside approximately 1.5 million out-of-hours consultations [7], placing these services at the forefront of early sepsis recognition and escalation.

Analysis of Hospital In-Patient Enquiry (HIPE) data (2020–2024) identified 68,644 sepsis admissions, of which 62.0% were community-acquired. However, HIPE data capture only admitted patients and therefore do not reflect the early trajectory of disease at emergency department (ED) presentation.

Patients presenting to ED with community-acquired acute infection remain poorly characterised in Irish research. Existing datasets do not capture patients who die in the ED prior to admission, near-miss cases treated and discharged, referral pathways (GP, OOH, paramedic, self-referral) and early physiological markers at presentation. Understanding these factors is essential to improve early detection, optimise resource allocation, and enhance patient outcomes. This study aims to characterise patients presenting to an Irish emergency department with community-acquired acute infection and to evaluate factors associated with adverse clinical outcomes.

### Study objectives

The primary objective of the study is to characterise patients presenting to the emergency department with community-acquired acute infection across eight predefined sampling periods.

The secondary objectives of the study are:

- To estimate the proportion of ED presentations attributable to community-acquired acute infection.
- To describe patterns of referral from general practice, GP out-of-hours services, paramedics, and self-presentation.
- To compare clinical presentations and source of referrals between weekday and weekend admissions.
- To determine the frequency of possible sepsis and identify factors associated with possible sepsis and adverse clinical outcomes.

## Methods

### Study design and setting

This study is a single-centre retrospective observational study of presentations to the emergency department of Mater Misericordiae University Hospital (MMUH), Dublin. The study used a cross-sectional approach to identify all presentations with community-acquired acute infection and an observational analytical component to examine factors associated with possible sepsis and clinical outcomes. MMUH is a large tertiary-level teaching hospital in Dublin with a high-volume urban emergency department that receives referrals from primary care, ambulance services, and self-presenting patients. It provides care to a predominantly adult population (aged ≥16 years) and does not provide maternity services. The hospital serves an urban catchment area with a significant proportion of socioeconomically deprived communities [8]. The study protocol and findings of the study will be reported in adherence to the recognised principles of the STROBE (Strengthening the Reporting of Observational Studies in Epidemiology) and RECORD (REporting of studies Conducted using Observational Routinely-collected health Data) statements.

### Sample size

To account for potential seasonal variation in infection patterns (e.g., winter respiratory surges), data will be collected from predefined 24-hour sampling periods across the year 2025: Winter – January 14th (Tue) & Jan 19th (Sun); Spring - April 14th (Mon) & Apr 20th (Easter Sun); Summer – July 14th (Mon) & July 20th (Sun); Autumn: October 14th (Tue) & Oct 19th (Sun). Sampling across both weekday and weekend periods is intended to improve representativeness while maintaining feasibility for detailed manual chart review. No formal sample size calculation was performed because all eligible presentations occurring during the predefined sampling periods were included. The study therefore represents a census of all eligible presentations rather than a sampled cohort.

### Inclusion criteria

All ED presentations during the predefined sampling periods will be screened. Patients meeting the case definition for community-acquired acute infection will form the study group. Total ED presentations will be used as the denominator to estimate the proportion of community-acquired infection presentations.

### Case Definition

Community-acquired acute infection was defined as documented clinical suspicion or diagnosis of infection recorded in ED triage documentation or emergency physician notes following manual review of the clinical record. Possible sepsis was defined as presentations in which the ED triage sepsis discriminator identified concern for sepsis, supported by documentation within the ED clinical record.

### Exclusion criteria

- Hospital-acquired infections developing ≥48 hours after hospital admission or clearly documented as hospital acquired were excluded. Community-acquired infection referred to infection present at, or before, emergency department presentation.

### Data collection and variables extracted

**Table:** 

**Demographic**	Age, Sex and Medical card status
**Clinical**	Presenting symptoms, NEWS score, suspected source of infection
**Process**	Referral source (GP, GP-OOH, paramedic, self-referral), ED sepsis treatment or completion of sepsis 6, IV antibiotics given (Yes/No)
**Outcomes**	Discharged from ED/ Ward admission/ Death in ED Time between ED admission and ward admission to inpatient admission, 30 day and 90 day mortality

Data will be extracted using a case report form (Table 1). Data collection is scheduled to begin in mid-May 2026 and will continue until December 2026. The study results are expected to be available in February 2027. Eligibility and data extraction will be independently undertaken by two reviewers using a standardised data collection form. Discrepancies will be resolved by discussion and consensus with a third reviewer.

**Table 1:** Case report form.

Section	Variable	Response
**Study Identification**	Study ID	
	Index ED attendance date	
	Repeat attendance	□ Yes □ No
	Hospital	
**Patient Demographics**	Age (years)	
	Sex	□ Male □ Female
	Eircode/Area of residence	
	Occupation	
	Medical card status	□ CAT1 □ CAT2 □ Other □ None
**Referral Information**	Referral pathway	□ GP □ GP-OOH □ Self □ Ambulance □ Other
	GP referral	□ Yes □ No
	Antibiotics prescribed by GP	□ Yes □ No □ Unknown
**ED Presentation**	Date and time of attendance	
	Presenting symptoms	
	Source of infection category	
	Organ/system involved	
	Mode of arrival	□ Own transport □ Fire Brigade Ambulance □ National Ambulance Service □ Other
	Triage category	□ Immediate □ Very Urgent □ Urgent □ Standard □ Non-Urgent
**Clinical Observations**	Temperature (°C)	
	Pulse (beats/min)	
	Systolic BP (mmHg)	
	Diastolic BP (mmHg)	
	Respiratory rate (/min)	
	Oxygen saturation (%)	
	Alert (AVPU/GCS)	
	CRP (mg/L)	
**Treatment in ED**	Antibiotics prescribed in ED	□ IV □ Oral □ None
**ED Time Metrics**	Time to triage (minutes)	
	Registration to clinician assessment (hours)	
	Attendance to admission (hours)	
	Attendance to discharge (minutes)	
	Attendance to discharge (hours)	
	ED registration to inpatient discharge (hours)	
**Disposition**	Disposition from ED	□ Admitted □ Discharged □ GP Follow-up □ OPD □ Left before completion □ Transfer □ Other
	Discharge date and time	
**Clinical Outcomes**	Reattendance to ED	□ Yes □ No
	Death during index episode	□ Yes □ No
	30-day mortality	□ Yes □ No
	90-day mortality	□ Yes □ No
	ED death	□ Yes □ No
	Discharged to another hospital	□ Yes □ No
	Inpatient death (surgical)	□ Yes □ No
	Inpatient transfer (surgical)	□ Yes □ No
**Comorbidities (Elixhauser)**	Myocardial infarction	□ Yes □ No
	Congestive heart failure	□ Yes □ No
	Arrhythmia	□ Yes □ No
	Valvular heart disease	□ Yes □ No
	Peripheral vascular disease	□ Yes □ No
	Hypertension	□ Yes □ No
	Stroke/paralysis	□ Yes □ No
	Other neurological disease	□ Yes □ No
	Chronic pulmonary disease	□ Yes □ No
	Diabetes mellitus	□ Yes □ No
	Hypothyroidism	□ Yes □ No
	Renal failure	□ Yes □ No
	Liver disease	□ Yes □ No
	Peptic ulcer disease	□ Yes □ No
	HIV/AIDS	□ Yes □ No
	Lymphoma	□ Yes □ No
	Metastatic cancer	□ Yes □ No
	Solid tumour	□ Yes □ No
	Rheumatoid/connective tissue disease	□ Yes □ No
	Coagulopathy	□ Yes □ No
	Obesity	□ Yes □ No
	Weight loss	□ Yes □ No
	Fluid and electrolyte disorders	□ Yes □ No
	Anaemia	□ Yes □ No
	Alcohol abuse	□ Yes □ No
	Drug abuse	□ Yes □ No
	Psychoses	□ Yes □ No
	Depression	□ Yes □ No
	Anxiety	□ Yes □ No
	ADHD	□ Yes □ No
	Smoking	□ Current □ Former □ Never
	Miscarriage (if applicable)	□ Yes □ No
	Diverticulitis	□ Yes □ No
	Urosepsis	□ Yes □ No
	Total number of comorbidities	

### Outcome measures

The primary outcomes measured are characteristics of possible sepsis presentations and referral pathways of community-acquired acute infections. The secondary outcomes include 30-day and 90-day mortality, hospital admission rate, sepsis pathway activation and referral sources.

### Data analysis

Statistical analysis will be performed using SPSS and Python notebook. Descriptive statistics will be used to summarise patient characteristics, referral pathways, and clinical outcomes. Categorical variables will be presented as frequencies and proportions, while continuous variables will be summarised using means with standard deviations or medians with interquartile ranges, depending on distribution. Associations between referral source and adverse outcomes will initially be explored using chi-square tests. Variables (age, sex, NEWS score, vital signs, C-Reactive Protein) associated with possible sepsis in univariable analyses together with clinically relevant covariates will be entered into an exploratory multivariable logistic regression model. Statistical significance will be set at p < 0.05. Missing data were reported for individual variables. Analyses were performed using complete-case analysis for each variable, with no imputation of missing values.

### Ethical considerations

Ethical approval was obtained from the Mater Misericordiae University Hospital (MMUH) Research Ethics Committee (REC Ref: 1/378/2584TMR). This study involves a retrospective review of existing clinical records only. Data will be extracted from patient records by authorised members of the research team (NR, EU, TB, AC), who may have access to identifiable information during the data collection process. However, no patient or clinician identifiers will be recorded in the study dataset. Data will be pseudonymised prior to analysis and stored in a secure format, with access restricted to authorised members of the research team. Mater Misericordiae University Hospital will act as the data controller, and all data will be stored and analysed within the institution. All electronic data will be maintained on encrypted, password-protected systems in compliance with GDPR regulations. As this study involves retrospective analysis of routinely collected clinical data and does not influence patient care, individual informed consent is not required and waived by the MMUH research ethics committee.

## Discussion

This study aims to provide a comprehensive evaluation of ED presentations with community-acquired acute infection in an Irish tertiary hospital. By examining all ED presentations during predefined sampling periods, the study seeks to estimate the proportion of patients presenting with community-acquired acute infection and to identify factors associated with adverse clinical outcomes.

A key strength of the study is its focus on the early phase of patient presentation, which is not captured in administrative datasets such as HIPE. By examining referral pathways, physiological markers at presentation, and early treatment decisions, the study will provide insights into how patients with community-acquired acute infection and possible sepsis enter and move through the healthcare system [9].

The study will also contribute to understanding the role of primary care and pre-hospital services in the recognition and escalation of severe infection [10]. Identifying patterns of referral and associated outcomes may inform improvements in early recognition strategies and communication between community services and emergency departments [11].

Several limitations should be considered. The study is conducted in a single tertiary centre, which may limit generalisability to other healthcare settings. The use of predefined 24-hour sampling periods, while pragmatic, may not capture all variability in infection patterns throughout the year. Additionally, the retrospective design relies on the completeness and accuracy of clinical documentation [12]. Despite these limitations, the findings are expected to provide valuable baseline data on the characteristics of community-acquired infection presentations in Irish emergency care during the predefined sampling periods.

The results may inform future prospective studies, sepsis pathway development, and national healthcare planning, particularly in relation to early recognition and management of community-acquired acute infection.
